# Communication skills supervisors’ monitoring of history-taking performance: an observational study on how doctors and non-doctors use cues to prepare feedback

**DOI:** 10.1186/s12909-019-1920-4

**Published:** 2020-02-06

**Authors:** Michaela Wagner-Menghin, Anique B. H. de Bruin, Jeroen J. G. van Merriënboer

**Affiliations:** 1 Medical University of Vienna, Teaching Center, Spitalgasse 23, A-1090 Vienna, Austria; 2 Maastricht University, School of Health Professions Education, P.O. Box 616, 6200 MD Maastricht, The Netherlands

**Keywords:** Communication skills, Feedback, Monitoring, Accurate self-judgements, Undergraduate medical education

## Abstract

**Background:**

Medical students need feedback to improve their patient-interviewing skills because self-monitoring is often inaccurate. Effective feedback should reveal any discrepancies between desired and observed performance (cognitive feedback) and indicate metacognitive cues which are diagnostic of performance (metacognitive feedback). We adapted a cue-utilization model to studying supervisors’ cue-usage when preparing feedback and compared doctors’ and non-doctors’ cue usage.

**Method:**

Twenty-one supervisors watched a video of a patient interview, choose scenes for feedback, and explained their selection. We applied content analysis to categorize and count cue-use frequency per communication pattern (structuring/facilitating) and scene performance rating (positive/negative) for both doctors and non-doctors.

**Results:**

Both groups used cognitive cues more often than metacognitive cues to explain their scene selection. Both groups also used metacognitive cues such as subjective feelings and mentalizing cues, but mainly the doctors mentioned ‘missing information’ as a cue. Compared to non-doctors, the doctors described more scenes showing negative performance and fewer scenes showing positive narrative-facilitating performance.

**Conclusions:**

Both groups are well able to communicate their observations and provide cognitive feedback on undergraduates’ interviewing skills. To improve their feedback, supervisors should be trained to also recognize metacognitive cues, such as subjective feelings and mentalizing cues, and learn how to convert both into metacognitive feedback.

## Background

Courses in medical communication tasks such as taking a patient’s history have been developed to allow students learning to communicate with patients early in the curriculum [1, 2]. Feedback from peers and/or supervisors on how well they conduct a patient interview is an essential instructional intervention [1–3], because students have been found to be moderately accurate at best at monitoring their own strengths and weaknesses [4–6]. Typically, supervisors are advised to communicate specific observations (=descriptive feedback, cognitive feedback, task-related feedback) including both positive and negative aspects of performance, rather than communicate judgements or evaluative adjectives that summarize behaviour as personality traits ([7] p 123). This is seen as a way of starting a conversation about what the learner was trying to achieve without hurting learner’s feelings ([7] p 125). This feedback strategy is supported by the learning sciences: Task-related negative feedback that does not touch the learners’ self-confidence has been found most effective in improving performance [8] and positive feedback encourages students to keep on working [9]. For example, instead of ‘you rushed through the first questions’, a supervisor should say ‘I saw that you phrased your first question as an open question, just like we practised, but I also saw that you gave the patient no time to answer. You asked another open question straight away.’

From a learning sciences perspective, cognitive feedback has been shown to boost performance better when accompanied by metacognitive feedback [10–12]. Effective metacognitive feedback stimulates a learner’s thinking about his own performance (=metacognitions on performance). It works by drawing learners’ attention towards relevant information available in the situation that is indicating good or weak performance. Because a learner’s metacognitive processes cannot be observed externally, providing metacognitive feedback requires prompting the students to share their thoughts, for example by giving a metacognitive prompt such as ‘What do you think of your first two questions?’ prior to giving descriptive feedback. The importance of eliciting learners’ metacognitions, together with their underlying knowledge and beliefs about the situation when giving feedback, has also been emphasized in connection with debriefing in anaesthesia [13].

Despite the importance of both cognitive and metacognitive feedback to driving students’ learning of interviewing skills, there is no established theoretical model to describe how supervisors observe, process, and integrate information on learners’ performance when preparing feedback. There is a model describing the cognitive processes related to rating students’ performance for summative assessment available [14], but it does not explain how supervisors come to their judgements. This is surprising given the ample evidence that when assessing students, judges’ observing, processing, and integrating of information does not always lead to converging judgements [14]. Instead, diverging judgements have been found to be caused by deviating views on integrating different aspects of performance in a single judgement [15, 16]. In connection with giving feedback on interviewing skills, diverging judgements have only recently been regarded as problematic. Critics fear that supervisors, who are not clinically active doctors, neglect the needs of clinical practice when giving feedback [17].

To learn more about the supervisors’ observation process we adapted a cognitive model on using information to generate self-judgements (=cue-utilization model [18]) to conduct an in-depth analysis of supervisors’ judgments. The model was used recently to describe how medical students interviewing a simulated patient monitor their behaviour [19]. We argue that this model can also be applied to model the supervisors’ observation process.

### Modelling history-taking related self-judgments and judgments

**Fig. 1:**
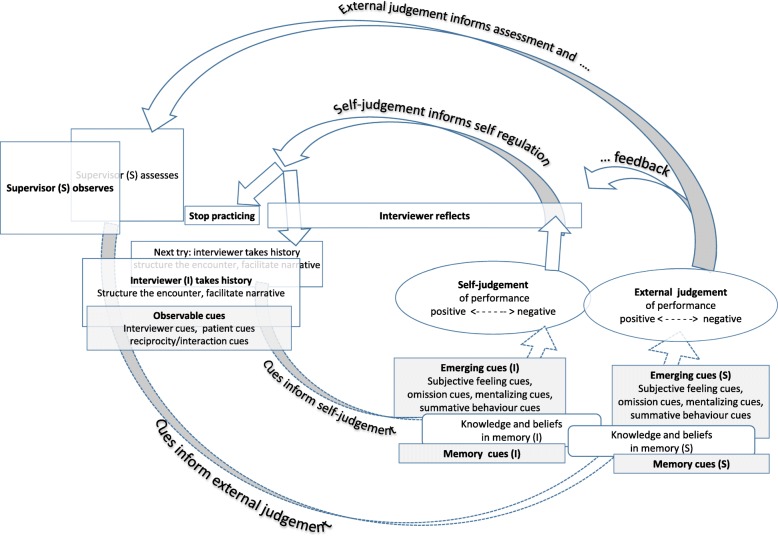
Self-regulating learning in patient interviews. Both the interviewer’s self-judgement (inner circle) and the supervisor’s external judgement (outer circle) rely on cues

Student interviewers seeking to self-regulate their learning need to evaluate what went well (positive self-judgement) and what did not go well (negative self-judgement; Fig. 1, inner circle, lower half) before being able to act based on these self-judgments (Fig. 1, inner circle, upper half circle). According to the cue-utilization model [18] a variety of cognitive and metacognitive cues can be perceived during history-taking, which inform these self-judgments. The cognitive cues include the patient’s verbal and nonverbal behaviour, the interviewer’s own behaviour, and how the interviewer coordinates his interaction with the patient (see box ‘Observable cues’ in Fig. 1: patient cues, interviewer cues, reciprocity/interaction cues). Also expectations stored in the interviewer’s memory (see box ‘Memory cues (I)’ in Fig. 1) are cognitive cues. The conscious processing of memory cues and observable cues is accompanied by unconscious processing which results in the emergence of several metacognitive cues. Subjective feeling cues are expressions of quality (e.g. ‘this went well’, ‘this ran fluently’ [20],); omission cues indicate that the goal of gathering information has not been met fully; mentalizing cues indicate that the interviewer interprets the patient’s experience in the situation (a typical metacognitive process in social interaction, called mentalizing [21]), and summative behaviour cues help to verbalize and summarize human behaviour (e.g., ‘I acted friendly and interested’). (See box ‘Emerging cues (I)’ in Fig. 1). However, as not all cognitive and metacognitive cues are diagnostic of task performance, self-judgement may be inaccurate and thus impede self-regulation (e.g. in the case of overestimating performance further practice to remediate a weakness will most likely not be undertaken [22]).

For example, a student being able to monitor accurately while interviewing a patient may notice: ‘I wanted to clarify the patient’s pain but it *did not run well’* (subjective feeling). I quickly asked a row of questions such as ‘*Does it hurt during walking?*’ and ‘*Does it hurt when you sit*? (observable cue/interviewer). The patient just answered ‘*yes*’ or ‘*no*’ (observable cue/patient) and I did not get rich information as the *questions were not phrased as open questions as they should have been* (memory cue). I also *forgot to explore the exact localisation and the quality of the pain* (omission cues), probably because I was so distracted by having to phrase questions quickly. In the end, when I summarized, the patient appeared to be rather frustrated (mentalizing cue), and although she was still *friendly* when saying goodbye (summative behaviour cue), I *got the impression she was not satisfied* (mentalizing cue) with the encounter. Whereas a student being not able to monitor accurately will probably notice: I wanted to clarify the patient’s pain and therefore asked about all the *topics to learn about situations in which a patient experiences pain, as given in the content guide (*memory cue). So I got all the information I needed. Unfortunately I *forgot to explore the exact localisation and the quality of the pain* (omission cues), which makes me look *unprofessional* (summative behaviour cue). Though in the end I summarized everything nicely (subjective feeling) and the patient was *friendly* when saying goodbye (summative behaviour cue).

In the current study we stress that also supervisors’ monitoring needs to be accurate to prepare helpful cognitive and metacognitive feedback. We thus extended the cue-utilization model by adding the supervisor’s processing related to observing students for either preparing feedback giving or judging in summative assessment (Fig. 1, outer circle). Then we used the model to hypothesize on how the supervisor’s professional background (doctor or non-doctor) influences their processing of observable cues, memory cues and emerging cues.

### Hypotheses

#### Hypothesis 1 - observable cues

Supervisors of communication-skills training are taught to focus on objectively observable behaviour when giving feedback [7] and when observing students for assessment purposes (14, see box ‘observable cues’ in Fig. 1: patient-cues, interviewer cues, reciprocity/interaction cues). A supervisor would thus prepare for giving feedback such as: I got the impression the student targeted at clarifying the patient’s pain. But he asked a row of closed questions such as ‘*Does it hurt during walking?*’ and ‘*Does it hurt when you sit*? very quickly. We thus hypothesize that irrespective of their professional background, supervisors prefer observable cues, that is, interviewer cues, patient cues, and reciprocity/interaction cues above all other cues.

#### Hypothesis 2 - memory cues

The supervisor needs to combine the observed cues with memory cues to form a judgement in order to select a scene for giving feedback (=processing skills [14]). Although differences in professional experience cause different knowledge and beliefs in memory of each individual, we assume an overlap in the memory structures relevant to communication-skills training. The knowledge about how to best phrase questions, for example, should be hold by all communication skills supervisors, leading to cognitions related to preparing feedback such as: ‘He asked a row *of closed questions* such as … ’ and to summative evaluation judgments indicating room for improvement in this aspect of patient communication (see box ‘Memory cues (S)’ in Fig. 1). We therefore hypothesize that doctors and non-doctors use memory cues in a similar way.

#### Hypothesis 3 - emerging cues

Emerging cues result from subconsciously combining information available in the situation with information from the supervisors’ memory (see box ‘Emerging cues (S)’ in Fig. 1). Different memory structures in doctors and non-doctors will cause different cues to emerge in consciousness. Both groups are trained to be attentive to their patient’s needs, but given that non-doctors have experience in establishing a therapeutic alliance for their work we assume that they will be more attentive to mentalizing cues and subjective feeling cues than doctors. Thus cognitions such as ‘This patient here seems not satisfied with the encounter (mentalizing cues), and I think it did not go well (subjective feeling cue), probably because of student’s way of asking … ’ are expected to be more common in non-doctors than in doctors. Both groups are trained to follow the structured content guide, but doctors are used to working with similar content in their professional practice thus we assume that they will be more attentive to omission cues than non-doctors.

#### Hypothesis 4 – summative behaviour cues

A special situation arises with the emerging summative behaviour cues, that is, using adjectives such as ‘friendly’ or ‘withdrawn’ spontaneously to summarize observed behaviour in interaction situations [23–25]. Supervisors are discouraged from using them in feedback as they do not help the student further improve their performance [7]. Therefore we assume that doctors and non-doctors similarly refrain from using them.

To summarize, supervisors observing a patient interview in order to select scenes for feedback are expected to prefer observable cues (Hypothesis 1) and memory cues (Hypothesis 2) and neglect verbal summative-behaviour descriptor cues (Hypothesis 4) irrespective of their professional background. Since different knowledge and beliefs in memory give rise to different emerging cues, doctors are assumed to use omission cues more often and non-doctors are assumed to use mentalizing cues/subjective feeling cues more often (Hypothesis 3). We developed a procedure to capture cue-processing in trained patient-communication supervisors and tested our hypotheses in a real-life setting.

## Method

This observational study seeks to describe influences of supervisors’ professional background on their processing of observable cues, memory cues and emerging cues in order to discuss their preparedness for giving cognitive and metacognitive feedback. It thus draws on material collected routinely in preparation for the Train-the-Trainer workshop for supervisors in second-year patient-communication training at the Medical University Vienna. A written assignment, focussed on reviewing and judging a video recording of a model history-taking performance, served as material for content analysis with deductive category application. We derived quantitative measures from this analysis. Workshop facilitators use the anonymized written materials as the starting point for group assignments and discussion during the Train-the-Trainer workshop.

### Participants

We approached 35 supervisors doing the Train-the-Trainer workshop that prepared them to teach patient-communication skills. Of this group, 10 doctors from five medical specialities and 7 non-doctors, including (clinical) psychologists, communication experts, and psychotherapists, consented to having their assignments analysed for this research.

### Materials and procedure

The video ‘Vomiting blood’ (6:19 min) [26] was used as standard stimulus material. In the video a senior physician performs a *focussed intake interview* in an emergency department with a male patient, portrayed by an actor. Besides the clinical details of the presenting symptom (vomiting blood) there are aspects of the patient’s perspective (bad prior experience with hospitals, patient did not want to come but was ordered to by his wife) and aspects of background history (former illnesses) to explore.

For analysis the video was divided into sections, each featuring a specific communication pattern, distinguished by the interviewer’s or patient’s utterances that indicate a shift in leading the conversation [27]. These shifts in conversation were identified by the first author and a research assistant who discussed each defined shift using the conversational model of Kurz, Silverman and Draper [7] and the model of Langewitz [26]. The communication pattern *structuring the encounter* was identified by interviewer’s utterances intended to organize the meeting (e.g., summarizing or transition statements) and retrieve information (e.g., opening question). The communication pattern *facilitating the patient’s narrative* was identified by four types of interviewer behaviour intended to enable patients to tell their story [1]: staying silent following a question [2], uttering verbal facilitators such as ‘hm’ [3], showing nonverbal facilitators such as ‘nodding’, and [4] actively repeating the patient’s utterances to emphasize attention and understanding. Table 1 gives time codes and durations of the identified sections, as well as the sections’ predominant communication pattern. To further describe the sections, the predominant communication challenge as defined by the conversational models, the supervisors’ predominant performance judgements (positive/negative), as well as numbers of supervisors (doctors/non-doctors) selecting a scene within each section for preparing feedback are included in Table 1.

**Table 1:** Communication pattern per section and number of supervisors selecting a scene within each section

Time code (Duration)	Communication pattern	Predominant communication challenge	Predominant performance judgment	Number of supervisors selecting scene (s)^a^		
				All *n* = 17	Doctors *n* = 10	Non-doctors *n* = 7
00:00–00:05 (5 s)	Structuring	Opening (hand shake)	negative	2	2	0
00:06–00:10 (5 s)	Structuring	ID check (using the computer)	negative & positive	8	5	3
00:11–00:30 (19 s)	Structuring	Introduce oneself	positive	4	2	2
00:11–00:30 (19 s)	Structuring	Set interview goals	negative & positive	3	2	1
00:11–00:30 (19 s)	Structuring	Set time frame (time pressure)	negative	13	7	6
00:31–00:33 (2 s)	Structuring	Opening question	positive	3	1	2
00:34–02:30 (1 min 56 s)	Facilitating	Biomedical details, listening, understanding, clarifying	very positive & negative	11	6	5
00:34–02:30 (1 min 56 s)	Facilitating	Patient’s perspective, listening, understanding, clarifying	positive & negative	10	3	7
02:31–03:25 (54 s)	Structuring	Summarizes and checks with patient, balancing important information	positive & negative	10	7	3
03:26–03:35 (9 s)	Structuring	Transition statement and opening question for topic ‘background information’	positive	3	2	1
03:36–05:05 (1 min 29 s)	Facilitating	Biomedical details, listening, understanding, clarifying	positive & negative	8	5	3
03:36–05:05 (1 min 29 s)	Facilitating	Patient’s perspective, listening, understanding, clarifying	positive & negative	1	0	1
05:06–05:23 (17 s)	Structuring	Summarizes and checks with patient, balancing important information	positive & negative	8	4	4
05:26–06:10 (44 s)	Structuring	Transition and opening ‘What’s next?’	positive	10	6	4
06:11–06:19 (9 s)	Structuring	End encounter	negative & positive	8	5	3

Notes: Structuring = communication pattern targeting at *structuring the encounter* (e.g. interviewer’s utterances such as summarizing or transition statements and information retrieval). Facilitating = communication pattern targeting at *facilitating the patient’s narrative* (e.g. Interviewer behaviour such as silence following a question, using verbal facilitators (‘hm’), showing nonverbal facilitators (‘nodding’), actively repeating the patient’s utterances to emphasize attention and understanding). Predominant Communication Challenge: determined based on the conversational model of Kurz, Silverman and Draper (2005). Predominant performance judgments: determined based on supervisors’ performance judgements (positive/negative) for their selected scenes

^a^All supervisors, except one, indicated only one scene within each section

Supervisors were instructed to watch the video as if they were observing a student in class, and prepare feedback by selecting scenes where they felt that something had happened that would influence the attainment of the communication goal either positively or negatively. The supervisors were instructed to rate their judgement on a seven-point scale with categories ranging from 1 = extremely negative to 7 = extremely positive, and were prompted to describe the reasoning behind it in writing (‘Briefly describe what is happening in the scene’ and ‘Explain why you consider this a positive or negative influence’). After completing the Train-the-Trainer workshop, the supervisors were invited to have their assignments included in the study. Upon giving written consent to participate, they were told that information about their professional background (doctor/non-doctor) would be retrieved. The study protocol was reviewed and approved by Medical University Vienna’s board for data protection (31.01.2015/02.02.2015).

### Analysis

#### Content analysis

Supervisors’ elaborations on their judgements were unitized in propositional units [28] with the structure *I observed/perceived X which I consider beneficial/detrimental because of Y.* The cue-utilization framework informed the coding dimension ‘types of cues used’ as specified previously [19] and included observable cues, memory cues and emerging cues, including summative behaviour cues. Table 2 presents definitions of cues and coding examples.

**Table 2:** Cues, their definition and examples

Cues	Definitions	Examples
Observable cues	What was done or said in the situation either by patient or by the interviewer that can also be seen or heard by an observer	–
Interviewer cues	–	I opened the information gathering by asking ‘…’
Reciprocity/Interaction cues	Interviewer cues and patient cues are related in a verbal statement.	I ask a series of closed questions, that is why the patient answers in a low voice with one worded answers.
Memory cues	Explicitly mentioning knowledge and beliefs when elaborating on a judgment. (Probe if the to be coded statement sounds like an answer in a knowledge test on communication skills).	Start the information gathering with an open question Explain medical terms using patient language, especially when patient looks puzzled
Emerging cues	Verbal elaborations including adjectives indicating that a comparison to an internal standard has taken place	–
Subjective feeling cues	Verbal elaborations using rather content-less, generic attributes.	This question appears to be inappropriate I have chosen a suitable transition statement This encounter does not run smoothly
Omission cues	Verbal elaborations targeting the covering of relevant content of patient’s story.	I forgot to explore about x & y.
Mentalizing cues	Verbal elaborations interpreting patient’s experience in the situation.	The patient feels ashamed having to talk about x & y. The patient is in an unpleasant situation.
Summative behaviour cues	Verbal elaborations using personality adjectives to summarize behaviour.	I appear to talk friendly. I ask very general questions.

#### Control variables

The analysis controlled for two factors related to scene selection. The first factor constituted the two major communication patterns that is, structuring the encounter and facilitating the patient’s narrative [17, 27] as both patterns require different behaviour from the interviewer. The second constituted dichotomizing the outcome of the performance judgement (positive versus negative) because feedback on positive performance and negative performance has been shown to differ in effectiveness (Kluger and DeNisis 1996, cited from 12).

#### Quantitative measures

The number and duration of the selected scenes were evaluated. Communication pattern frequencies and performance judgement outcomes were counted, as well as cue-usage frequencies. A chi-square test was used to check for differences between doctors and non-doctors, and standardized residuals were used to describe the inclination to prefer rating scenes positively or negatively for both communication patterns. Effects were labelled small, medium, and large according to conventions (*d* = 0.2/ = 0.1: small, *d* = 0.5/*w*=0.3: medium, *d* = 0.8/*w*=0.5: large) [29]. To further describe differences in cue usage we used the *c-*index (ATLAS.ti GmbH 1993–2014) that normalizes the co-occurrence of using cues together with communication patterns and positive/negative judgements. The *c*-index varies between 0 and 1, where 0 indicates that the two variables never co-occur and 1 that they do co-occur in all instances. The degree of co-occurrence was labelled as follows: low degree (*c* < 0.25; meaning both codes are used in <25% of the cases), medium degree (*c* > 0.25 and *c* < 0.75), and high degree (*c* > 0.75). Atlas.ti, Version 7 (Atlas.ti GmbH, Berlin/Germany) was used for content coding and counting frequencies of code occurrence.

## Results

### Unitizing

Supervisors selected a total of 98 scenes relevant for further consideration. Median length of selected scenes was 30 s (min = 3 s, max = 174 s). It can be assumed that the numerical distribution (*U* = 49, exact *p* = .556) and duration (*U* = 2352; asymptotic *p* = .093) of selected scenes is equal between the two groups. Sequences longer than 2 min often covered more than one communication pattern and were split for analysis, resulting in 109 units for analysis. Judgements covering the whole encounter (*n* = 2) were excluded.

### Control variables

A chi-square test of independence was calculated comparing the frequencies of judging different communication patterns for doctors and non-doctors as either positive or negative. A significant interaction was found (χ^2^ = 9.960 [3]; *p* = .019). Doctors gave negative judgements more often for structuring the encounter and facilitating the narrative, as illustrated by the positive standardized residuals (0.8 and 1.0). Doctors and non-doctors identified positive judgements for scenes of structuring the encounter equally often (standardized residuals equal 0 for both groups). Doctors gave positive judgements for scenes illustrating facilitating the narrative less often than non-doctors, as indicated by the low value of the standardized residual for doctors (−1.6) and the high value (1.9) for non-doctors (see Table 3). Thus, to test the hypotheses, cue-usage frequencies for doctors and non-doctors were tabulated for all communication pattern and positive/negative judgement combinations (see Table 4).

**Table 3:** Judging scenes positively or negatively from different communication patterns: frequencies and standardized residuals

	Doctors	Non-doctors
Structuring the encounter & negative judgement	18 (0.8)	8 (−0.9)
Structuring the encounter & positive judgement	27 (0.0)	20 (0.0)
Facilitating narrative & negative judgement	11 (1.0)	3 (−1.2)
Facilitating narrative & positive judgement	7 (−1.6)	15 (1.9)
Number of scenes (*N* = 109)	*n* = 63	*n* = 46
*Effect size; Statistics (df); p-values*	*ω = 0.33;* χ^2^ = 9.960 (3); *p* = 0.019

**Table 4:** Video of patient vomiting blood: Differences in using cues when judging scenes devoted to structuring the encounter and facilitating the patient’s narrative. Frequencies (*c*-index)

	Doctors					Non-doctors				
	Total	Structuring the encounter		Facilitating patient’s narrative		Total	Structuring the encounter		Facilitating patient’s narrative	
		Judgements		Judgements			Judgements		Judgements	
		Negative	Positive	Negative	Positive		Negative	Positive	Negative	Positive
Number of judgements	63	18	27	11	7	46	8	20	3	15
Used cue as unit of analysis (N=)	147	46	61	24	16	97	16	44	7	30
Observable cues										
Interviewer cues	49	15 **(0.29)**	26 **(0.52)**	6 (0.11)^	2	26	7 **(0.26)**	14 **(0.44)**	2	3
Reciprocity/interaction cues	13	3 (0.11)^	1	4 (0.20)^	5 **(0.33)**	18	1	5 (0.15)^	1	11 **(0.50)**
Memory cues	23	12 **(0.41)**	6 (0.14)^	5 (0.17)^	0	14	7 **(0.47)**	2	3 (0.21)^	2
Emerging cues										
Subjective feeling cues	33	10 (0.24)^	14 **(0.30)**	4 (0.10)^	5 (0.14)^	13	1	10 **(0.43)**	0	2
Omission cues	6	0	2	4 **(0.31)**	0	1	0	1	0	0
Mentalizing cues	14	5 (0.19)^	7 (0.21^)	0	2 (0.11)^	14	0	6 (0.21)^	1	7 **(0.32)**
Summative behaviour cues	8	1	5 (0.17^)	0	2 (0.15)^	10	0	6 **(0.25)**	0	4 (0.19)^

Notes: *c*-index: ^ indicates low degree of co-occurrence (*c* < 0.25; meaning both codes are used in < 25% of the cases), **bold print** indicates medium degree of co-occurrence (c > 0.25 and < 0.75), and high degree of co-occurrence (*c* > 0.75) did not occur, values <0.10 are not given

### Doctors’ & non-doctors’ Cue usage when judging

#### Observable cues – hypothesis 1

When selecting scenes devoted to structuring the encounter, both groups often used interviewer cues for negative and positive judgements. When selecting scenes related to facilitating the patient’s narrative, both groups did not use interviewer cues for either type of judgements (Table 4, row ‘interviewer cues’, four shaded cells with medium *c*-indices as compared to four unshaded cells with low/very low c-indices). Doctors use reciprocity cues quite often when positively judging facilitating the narrative, but the same is true for non-doctors (both *c*-indices show a medium degree of co-occurrence; row labelled reciprocity cues, two shaded cells). The findings for observable cues support Hypothesis 1, which states that both groups use observable cues in a similar way. However, doctors sometimes also use reciprocity cues when judging scenes negatively (both *c*-indices show low degree of co-occurrence), whereas non-doctors use reciprocity cues only for positive judgements (low degree of co-occurrence). These findings are not in line with Hypothesis 1.

#### Memory cues - hypothesis 2

Both groups use memory cues when judging scenes negatively. *C*-indices for structuring the encounter indicate a medium degree of co-occurrence and a low degree of co-occurrence for facilitating the patient’s narrative for both groups (Table 4, row ‘memory cues’). This finding supports Hypothesis 2 which assumed a similar usage of memory cues for doctors and non-doctors. Doctors, but not non-doctors, also use memory cues for judging scenes related to structuring the encounter positively.

#### Emerging cues - hypothesis 3

Doctors judging facilitating the patient’s narrative negatively use omission cues nearly exclusively (medium degree of co-occurrence, *c*-index = 0.31), thus partly supporting Hypothesis 3. Non-doctors use mentalizing cues more often when judging facilitating patient’s narrative positively (medium degree of co-occurrence, *c*-index = 0.32); doctors use them less often for three out of the four types of judgement (low degree of co-occurrence or no co-occurrence) thus also supporting Hypothesis 3. Against expectations, doctors rely on subjective feeling cues for all four types of judgement (low/medium degree of co-occurrence, see Table 4, row ‘subjective feeling cues’). Non-doctors, on the other hand, use subjective feeling cues only for positive judgements of structuring the encounter (medium degree of co-occurrence, *c*-index = 0.43).

#### Verbal summative behavior cues - hypothesis 4

Both groups seldom use summative behaviour cues, but when they are used, then nearly exclusively for positive judgements (low/medium degree of co-occurrence, c-indices = 0.17, 015, and 0.25, 0.19, Table 4 summative behaviour cues) and not for negative judgements. This supports Hypothesis 4 for similar usage of this type of cue.

## Discussion

We set out to investigate the preparedness of doctors and non-doctors to give cognitive and metacognitive feedback after observing a student conduct a patient interview. To model information processing when supervisors select scenes for feedback, we adapted the cognitive cue-utilization model and developed a research procedure to capture the cue usage.

Applying the model to hypothesize on similarities and differences in cue use, we found that both groups similarly prefer observable cues and memory cues above other cues on which to base their selection and judgement of scenes. Both groups rely heavily on the interviewer’s observable behaviour to identify structuring the encounter scenes. In addition, when talking about positive aspects related to facilitating the patient’s narrative, both groups utilize observable cues indicating reciprocity in the interaction between interviewer and patient. Hypothesis 1, on similar use of observable information irrespective of professional group, is thus confirmed. Due to the low absolute numbers of used reciprocity cues the impression arises that both professional groups focus more on interviewer behaviour than on how the interviewer interacts with the patient. Further research will have to establish if this also translates to focussing on interviewer behaviour in giving cognitive feedback and whether this type of feedback improves patient-oriented interviewing skills. Memory cues were often used when supervisors judged structuring the encounter negatively, supporting Hypothesis 2, on both groups sharing knowledge and beliefs related to their role as supervisors. Both groups also refrain from using the emerging summative behaviour cues thus confirming the similar usage of this type of cue (Hypothesis 4). When such a cue is used, it is mostly combined with positive judgements. Refraining from communicating judgements is in line with recommendations in the literature stating that interviewers should have the opportunity to make their own inferences (=self-judgments) first and hear someone else’s inferences (=judgements) only later [7].

However, as illustrated by the cue-utilization model, interpretations or inferences that emerge as cues are an important part of metacognitive processing. They cannot be completely suppressed and thus influence not only the student’s self-judgement of performance but also the supervisor’s selection of scenes for feedback.

Given their relatedness to memory, we expected doctors rather than non-doctors to use omission cues and that non-doctors would rather use mentalizing cues and subjective feelings than doctors. We found that doctors mainly used the omission cue, thus supporting Hypothesis 3, with negative judgements of facilitating the patient’s narrative. But other findings also contradict Hypothesis 3: Doctors included subjective feeling cues and mentalizing cues in all sorts of judgements, whereas non-doctors used these cues only with positive judgements.

Summarizing our results, both groups of supervisors rely similarly on observable interviewer behaviour cues and memory cues, which meaningfully inform cognitive feedback on interviewer performance.

Focussing on observable cues, supervisors seem to neglect patient cues when selecting scenes for feedback. However, this is where the importance of – the more judgemental – emerging cues comes into play. Both groups at least partly rely on their own subjective feelings and their interpretation of the patient’s experience when selecting scenes for feedback. For negative judgments, supervisors shy away from indicating their emerging inferences about the situation as the source of their judgments in an attempt to comply with the descriptive, non-judgemental approach to preparing feedback. But as they do indicate emerging inferences as the source of their positive judgments, it is plausible to assume that they also experience emerging inferences for negative judgments. Our results provide empirical evidence that ‘*despite a desire to seem non-judgmental, hints of one’s view often leak* [ …]. ’ ( [13], p., 368), because it is not possible to provide feedback to a situation without having it judged previously.

Basically, experiencing the emerging of inferences puts communication skills supervisors in a good position to initiate the process of giving metacognitive feedback. Thus supervisors should not only be trained to give descriptive cognitive feedback, but also in how to give metacognitive feedback prompts. The idea to have supervisors utilize their emerging inferences as a starting point for ‘*Debriefing with good judgment*’ ( [13], p., 396) has been previously suggested related to anaesthesia simulation training. In such training, supervisors are encouraged to communicate emerging inferences about the situation such as ‘*It looked to me like it was confusing*’ ( [13], p., 372) to establish agreement about a problematic result during the simulation. Only then observable behaviour is communicated and interpreted by the supervisor (=advocacy) followed by an inquiry about learner’s underlying knowledge and beliefs.

We suggest to adapt a similar approach when giving feedback on patient communication skills. Supervisors should initiate metacognitive feedback prior to giving cognitive feedback following the three-step approach depicted in Table 5.

**Table 5:** A three-step approach to giving cognitive and metacognitive feedback in history taking

Step	Description of step
1 Notice your emerging inferences & link them to behaviour	As a supervisor be aware of the observed patient’s experience and how you yourself experienced the situation. Be also aware of underlying observations of behaviour that caused your experience.
2 Metacognitive Feedback	Phrase a mentalizing prompt drawing the student’s attention to the patient’s experience. - Listen to student’s answer Describe how you interpret patient’s experience (or have other observers describe their interpretations) to stimulate comparison between student’s interpretation, external interpretations and knowledge and beliefs about favourable patient experiences.
3 Cognitive feedback	If necessary: Phrase an observation prompt drawing the student’s attention to the underlying behavioural issues. - Listen to student’s answer Describe the observed behaviour (or have other observers describe their observations) to stimulate comparison between observed behaviour and knowledge and beliefs about effective behaviour.

Supervisors need to notice their emerging inferences and their underlying behavioural source (Step1, Table 5) but withhold this information. Instead they need to convert their emerging inferences into neutral metacognitive prompts (Step 2, Table 5) to initiate a student’s reflection on the issue. Dependent on the student’s answer to the prompt a description of supervisor’s emerging inferences is helpful to stimulate use of relevant information in memory and make the transition to the cognitive feedback phase (Step 3, Table 5). Here, an observation prompt might be helpful in stimulating student’s patient observation, and describing observations for the student is again helpful to stimulate the use of relevant information in memory.

For example, a supervisor experiencing patient’s hesitation (mentalizing cue) and silence concerning the onset of symptoms (reciprocity cue) may link these experiences to student’s asking questions without leaving time for the patient to answer (interviewer cue) (Step 1). Typically the supervisor now would give descriptive performance feedback such as: ‘When you asked about the onset of the symptoms I saw your patient say two words very slowly before stopping and looking away from you.’ We propose instead to first initiate metacognitive feedback (Step 2) by converting this interpretation of patient behaviour into a general metacognitive prompt such as: ‘Were there any moments in the encounter when your patient felt uncomfortable?’ Or a specific prompt such as: ‘How easy or hard do you think it was for the patient to answer your question about the onset of the symptoms?’ Asking such questions, the supervisor draws the student’s attention to what the patient has experienced in the encounter. Only then cognitive feedback can be given (Step 3). This tactic is assumed to be effective in two ways. First, if students can be reminded of the respective incidents, or can view a video recording of the encounter, they can benefit by reflecting on how they could change their behaviour. Second, there is potential benefit even if the student cannot recall the situation, because now they know that paying attention to patients’ experience is a good idea.

Our study has three limitations which future studies may address. First, our video showed a senior doctor interviewing a patient. Supervisors may have reacted differently if they had seen a student in the role of interviewer. Lacking the availability of a published video of a student, we adopted the video actually used in our Train-the-Trainer course. Second, we instructed supervisors to select a scene, and then indicate and explain their judgement. Thus we captured how they justify their selection of scenes as the basis for feedback, but we did not directly capture their feedback-giving behaviour. Future studies may consider asking supervisors to write down the feedback they would like to give to the interviewer. A third limitation is that we had little opportunity to observe cue usage with negative judgements in non-doctors, because this group was smaller than the group of doctors and they rarely gave negative judgements. The lack of negative judgements might be related to the video model introducing himself as a senior doctor; again the response might be different if the stimulus material had shown a student.

## Conclusion

Using dedicated theory to hypothesize on supervisors’ cognitive process when preparing to give feedback, helped us gain insight into how different professional groups use cues to select relevant scenes. We see it as a strong point that we were able to pursue a dedicated theory-based approach in measuring how supervisors use cues. Both groups predominantly use observable cues to identify positive and negative performance. When elaborating on negative performance, both groups often refer to memory cues. Our main conclusion is that, as recommended, both groups adhere to the principle of giving descriptive feedback [7]. They prefer utilizing observable behaviour and memory cues, such as checklist entries for structuring the encounter, when justifying their scene selection. And they refrain from using summative behaviour cues when justifying their selection. We thus consider doctors and non-doctors both well prepared to give cognitive feedback. Especially when describing positive performance, both professional groups also mention cues showing more judgemental elements than descriptive elements, such as summative behaviour cues and subjective feelings. This emphasizes that identifying discrepancies between actual and expected patient-communication performance is a complex process whose success cannot be informed by observable cues only. As such giving feedback necessarily includes judgemental or evaluative elements. However, these judgments should be ‘*good judgments* [13]’ insofar as they should be closely linked to the situation at hand. Within the proposed cue-utilisation model we make an important contribution to the research literature, by more closely defining ‘good judgments’ in the domain of patient-communication for history taking. The emerging cues are ‘good judgments’ as long as they can still be linked to observable cues and knowledge and beliefs in memory. The value of using observed behaviour as a starting point for cognitive feedback has long been recognized in communication-skills training. Yet, the theory on using cues for evaluating performance, as well as our findings, call for explicit recognition of the value of inferences or judgements in giving metacognitive feedback.

## Data Availability

The datasets used and/or analysed during the current study are available from the corresponding author on reasonable request. Please note that raw data for the content analysis is in German.

## Abbreviations

I: Interviewer
S: Supervisor
